# An Ionic Limit to Life in the Deep Subsurface

**DOI:** 10.3389/fmicb.2019.00426

**Published:** 2019-03-12

**Authors:** Samuel J. Payler, Jennifer F. Biddle, Barbara Sherwood Lollar, Mark G. Fox-Powell, Thomas Edwards, Bryne T. Ngwenya, Sean M. Paling, Charles S. Cockell

**Affiliations:** ^1^UK Centre for Astrobiology, School of Physics and Astronomy, University of Edinburgh, Edinburgh, United Kingdom; ^2^College of Earth, Ocean, and Environment, University of Delaware, Lewes, DE, United States; ^3^Department of Earth Sciences, Toronto, ON, Canada; ^4^School of Earth and Environmental Sciences, University of St. Andrews, St. Andrews, United Kingdom; ^5^Israel Chemicals Ltd. Group, Whitby, United Kingdom; ^6^School of Geosciences, Kings Buildings, University of Edinburgh, Edinburgh, United Kingdom; ^7^Boulby Underground Science Facility, Science and Technology Facilities Council, Swindon, United Kingdom

**Keywords:** evaporite, salt, habitability, astrobiology, subsurface

## Abstract

The physical and chemical factors that can limit or prevent microbial growth in the deep subsurface are not well defined. Brines from an evaporite sequence were sampled in the Boulby Mine, United Kingdom between 800 and 1300 m depth. Ionic, hydrogen and oxygen isotopic composition were used to identify two brine sources, an aquifer situated in strata overlying the mine, and another ambiguous source distinct from the regional groundwater. The ability of the brines to support microbial replication was tested with culturing experiments using a diversity of inocula. The examined brines were found to be permissive for growth, except one. Testing this brine’s physicochemical properties showed it to have low water activity and to be chaotropic, which we attribute to the high concentration of magnesium and chloride ions. Metagenomic sequencing of the brines that supported growth showed their microbial communities to be similar to each other and comparable to those found in other hypersaline environments. These data show that solutions high in dissolved ions can shape the microbial diversity of the continental deep subsurface biosphere. Furthermore, under certain circumstances, complex brines can establish a hard limit to microbial replication in the deep biosphere, highlighting the potential for subsurface uninhabitable aqueous environments at depths far shallower than a geothermally-defined limit to life.

## Introduction

Several variables are often discussed when considering the limits to life in the deep subsurface, including temperature, pressure and limited energy and carbon sources. One aspect less commonly examined is the impact of dissolved ions in limiting microbial replication. Interactions between dissolved ionic species are extremely complex, particularly in natural brines, and vary with the ions present. For example, hydration shells around a chloride ion in a solution behave differently when Mg^2+^ is present instead of Na^+^ (Ohtaki and Radnai, 1993). The effect of such interactions on brine habitability are only just beginning to be understood. Extremes in a range of different physicochemical stressors brought about by dissolved ions have been demonstrated to preclude microbial propagation. These stressors include water activity (Tosca et al., 2008), chaotropicity (Hallsworth et al., 2007), and ionic strength (Fox-Powell et al., 2016). Recent work by Steinle et al. (2018) highlights how little we know about these boundaries, showing that microbial propagation might be possible in conditions previously thought to be uninhabitable.

The ionic composition of deep subsurface waters is particularly important in evaporite deposits where the dissolution of salt minerals can lead to fluids becoming highly concentrated in ions. Evaporites are ubiquitous in the deep subsurface, underlying 35–40% of the US with some sequences being hundreds of meters thick (Davies and LeGrand, 1972). This makes them a significant potential habitat in the terrestrial subsurface. Furthermore, evaporite deposits are widely exploited as a mineral resource, hence a better understanding of geochemical and biological processes occurring within them will carry important economic implications.

Little is known about the variability in brine composition in evaporite deposits and their impact on deep subsurface microbiology. Work carried out on the microbial communities in evaporite sequences has generally focused on halite-rich environments. In these environments both halophilic archaea and bacteria have been identified, with the order Halobacteriales being a prominent feature (e.g., Denner et al., 1994; Vreeland et al., 2000; Radax et al., 2001; Fish et al., 2002; Stan-Lotter et al., 2002; Mormile et al., 2003; Gruber et al., 2004). Gypsum horizons found 200 m below the Dead Sea were also found to contain Halobacteriales (Thomas et al., 2014). Whilst the literature on deep subsurface halite-rich environments is limited, it is worth noting that evaporite sequences consist of many soluble salt minerals assemblages that include no or little halite. Therefore, waters through the entire evaporite sequence will be more chemically diverse than those typically encountered in the halite dominated sections.

To examine the impact that brine ionic composition has on deep subsurface communities, we sampled a range of deep subsurface brines in Boulby Mine, a working potash mine located in Cleveland, northeast England. Geochemical and physicochemical assays were used to determine the chemical composition and its impact on the physicochemical state of the brines. Culturing experiments were used to test the ability of brines to support microbial replication. Those brines shown to be habitable were subject to metagenomic sequencing to investigate their microbial communities and compare them to other hypersaline communities.

## Materials and Methods

### Site Description

Boulby Mine (54.5561°N, 0.8216°W) provides direct access to an extensive subsurface evaporite – a Permian Zechstein evaporite sequence, the remains of a large epeiric sea which once spread from the east coast of the United Kingdom to Eastern Europe. Geologically, the mine is situated in the Cleveland Basin at the western margin of the North Sea Basin (Talbot et al., 1982). The sequence at Boulby is composed of repeating cycles of dolomite, anhydrite, halite, carnallite and shale (Davison, 2009), overlain by a ∼200–300 m thick aquifer in the Triassic aged Sherwood Sandstone. The mine is constructed primarily in a 50 ± 15 m thick halite layer at ∼1.1 km depth, overlain by a potash seam (sylvinite).

### Sampling Methods

**FIGURE 1:**
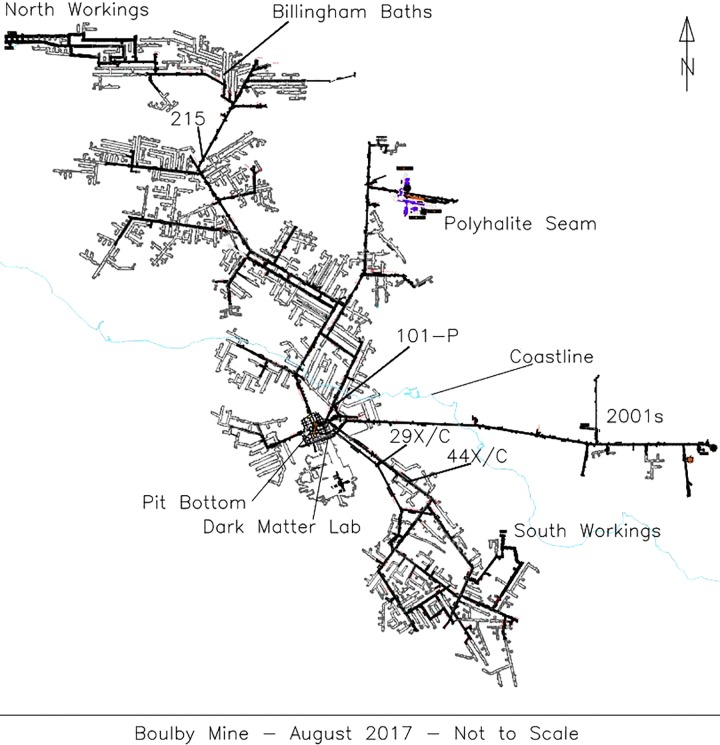
A schematic of Boulby mine in August 2017 with the locations of the brine seeps collected during this study. The coastline is outlined in light blue.

**FIGURE 2:**
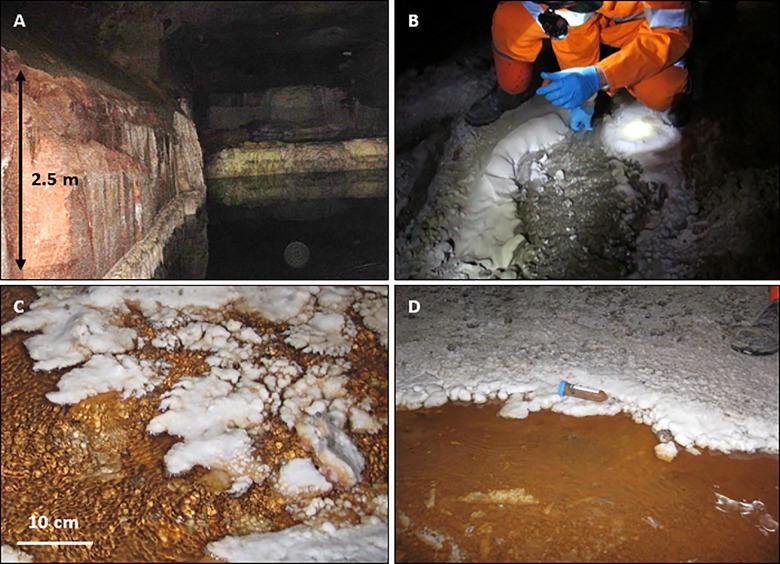
Contextual photographs of two brine pools sampled during this study. **(A)** Billingham Baths brine pool. **(B)** Brines flowing into brine pool at Billingham Baths (person for scale). **(C)** Close up of brine flows at 215. **(D)** Brine flow at 215. Red color is from the clay minerals present. Fifty microliter falcon tube for scale.

Five brine flows were sampled from Boulby between February 2013 and January 2015, found at depths between 800 m and 1.3 km (locations on Figure 1). These consisted of two pressurized brine seeps (Brine 44XC and 29XC), sampled directly from fractures in the evaporite (using taps that had been in place for < 1 year) and three recently ponded brine pools (Billingham Baths, Brine 215 and 101-P) (Figure 2). The two brine seeps allowed examination of native evaporite-hosted subsurface communities, whilst the brine pools were targeted to find out what becomes established in more permanent bodies of water after exposure to ambient conditions.

Brines to be analyzed for ionic composition, chaotropicity and water activity were filtered through 0.22 μm filters into sterile 50 mL polypropylene centrifuge tubes using a sterile syringe and placed in a cooler box with ice packs. For cation analysis, 1 M nitric acid was added (to a concentration of 1.5 mL/L), reducing the pH to prevent cation precipitation (Jackson, 2000). Samples to be analyzed for Total Inorganic Carbon/Total Organic Carbon (TIC/TOC) were collected in furnaced glass bottles, with the tops covered with furnaced aluminum foil and then sealed with plastic screw-cap lids.

Brines used for aerobic culturing (both as an inoculum and as a medium) and DNA extraction were collected in sterile 50 mL centrifuge tubes. Those to be used for anaerobic culturing were added to sealed sterile anaerobic serum bottles containing L-cysteine-HCl (as a reducing agent) to a final concentration of 0.8 mM. They were all then placed in a cooler box with ice packs to reduce microbial activity. Once at the surface, all aerobic and anaerobic samples were stored at 4°C and those for DNA extraction at −80°C.

For oxygen and hydrogen isotopes, samples were collected in 500 mL screw cap polypropylene Nalgene bottles, with no headspace. These bottles have been shown to maintain the original δ^18^O and δ^2^H values of water stored in them for at least 1 year (Spangenberg, 2012). Samples were stored overnight in a dark cold room at 4°C and shipped within 24 h to the University of Toronto, Canada.

### Brine Geochemical Analysis

Oxidation-reduction potential (ORP), pH and temperature were recorded *in situ* using a Myron Ultrameter II^TM^ 6Psi (Carlsbad, CA, United States). All pH measurements were corrected for temperature and salinity, and were calibrated using standards provided with the instrument. The Ultrameter II has an inbuilt electronic ORP and temperature calibration. All measurements were repeated three times, and the sensor rinsed with distilled water and dried between samples. DO readings were taken from the brine pools using a Spectrum Dissolved Oxygen Meter (model 407510) (Bridgend, Wales, United Kingdom). Measurements were calibrated and corrected for salinity and temperature on the instrument as described in the instrument manual. The set-up of the taps in the mine meant that brines 29XC and 44XC had to be exposed to the air during sample collection, making it impossible to get an accurate reading of DO without a major redesign of the pumping system. ORP measurements were also limited by this problem, but since they tend to react more slowly to environmental changes, they were recorded with this limitation in mind. Pressure measurements for 29XC and 44XC were provided by the mine from their regular monitoring 2 days before sampling was carried out.

ICP-OES (Inductively coupled plasma – optical emission spectrometry) analyses were carried out on a Perkin Elmer (Waltham, MA, United States) Optima 5300 DV ICP-OES instrument by the School of Chemistry at University of Edinburgh. Brines had to be diluted with 18.2 M Ω-cm distilled water from a Thermo Fisher Scientific (Waltham, MA, United States) Barnstead NanoPure system by factors of 10^2^–10^3^ to match the emission intensity of the standards. After dilution, Fe was the only element measured in the brines to fall below detection limits (necessary dilution of brines before analysis changed the Fe detection limit in the original brine from approximately 0.005–5 mg/l). The wavelengths used to interrogate specific ions were: Na^+^ 330.237, 588.995, 589.592 nm; K^+^, 766.490 nm; Mg^2+^, 279.077, 280.271, 285.213 nm; Ca^2+^, 315.887 nm, 317.933, 393.366 nm, 396.847 nm; Fe 239.562 nm. When using more than one wavelength per element, the wavelength with the highest *r*^2^-value was chosen to calculate the concentration of that ion in the sample.

${\text{NO}}_{3}^{-}$, ${\text{SO}}_{3}^{2-}$, Cl^−^, and Br^−^ were measured using Ion Exchange Chromatography (IEC). IEC analysis was carried out by the University of Sheffield by the Groundwater Protection and Restoration Group with a Dionex (now Thermo Fisher) ICS-3000. Samples were filtered through a 0.45 μm pore diameter membrane and diluted 10^3^ times with UHQ (Ultra High Quality) water before analysis. This brought them below the detection limits for ${\text{NO}}_{3}^{-}$ for all the brines except 44XC. The column used was an AS18 2 × 250 mm and 34 mM KOH at 0.25 mL/min was used as the eluent. Particulate and organic contaminate removal was achieved using a guard column with identical packing as the analytical column. The conductivity signal from ions in the eluent was suppressed with a micro-membrane suppresser between the analytical column and the conductivity detector. TOC/TIC was also carried out in the same laboratory in Sheffield using standard procedures on a Shimadzu TOC-V Series Total Organic Carbon Analyzer.

The δ^2^H analyses were performed via manganese reduction at 900°C using a method modified from Coleman et al. (1982). The δ^18^O analyses were performed by the CO_2_ equilibration method of Epstein et al. (1953). Reproducibility on duplicate analyses are ± 0.4‰, and ± 0.1‰ with respect to V-SMOW for δ^2^H and δ^18^O, respectively (see Table 1 for summary of geochemistry results).

**Table 1:** Brine geochemistry and physicochemical characteristics from all the brines collect from Boulby Mine, United Kingdom.

Ions (mg/l)	Billingham	215	29XC	44XC	101-P
F^−^	b.d.	b.d.	b.d.	b.d.	b.d.
Cl^−^	191,250	197,250	209,639	175,002	159,347
${\text{NO}}_{3}^{-}$	<100	<100	<100	560	<100
${\text{SO}}_{4}^{-2}$	2045	1980	1267	3836	2545
Ca^+^	121	112	1315	1650	1274
K^+^	971	34	66,863	4758	958
Mg^+2^	128	80	14,877	12,384	34,604
Fe	<50	<50	<50	<50	<50
Na^+^	117,396	120,784	9174	84,357	61,659
Br^−^	<0.1	<0.1	2463	523	233
pH	7.12	7.25	4.96	5.15	3.42
Temperature (°C)	29.5	32.4	35.5	35.7	33.1
δ^18^O	−7.5	−7.4	−7.3	−7.2	−5.2
δ^2^H	−48	−48	−40.5	−41.8	−37.8
Water activity (a_w_)	0.727	0.711	0.730	0.742	0.566
Chao/kosmotropicity as change in heat capacity (kJ kg^−1^)	n.a.	−64.08	−91.56	−70.98	133.55
Ionic strength (mol/L)	5.32	5.38	5.32	5.55	6.56

${\mathrm{SO}}_{4}^{2-}$, *Cl^−^, and Br^−^.*

### Culture-Dependent Analysis of Brine Ability to Support Microbial Growth

Culturing methods were used to determine which brines were habitable. To achieve this, an initial round of enrichment experiments was used to test which brines would support the growth of microorganisms from various inocula. Environmental brine media were created by filtering 500 mL of the five brines using 0.45 μm syringe-driven polycarbonate filter units (Merck Millipore; Burlington, MA, United States). Two sets of carbon sources were added to the brines as powders: one media containing just yeast extract (4 g/L), and the other a mixture of sodium pyruvate and casamino acids (1 and 2 g/L, respectively). The brines were sterilized in an autoclave (121°C for 20 min) and a 50 mL aliquot transferred to sterile glass conical flasks and topped with foam bungs wrapped in tin foil. An artificial media was also used to enrich organisms, prepared with the following recipe: NaCl 250 g/L, KCl 2 g/L and tryptic soy broth 25 g/L or nutrient broth 19 g/L (both Difco Laboratories, now Thermo Fisher) both balanced to pH 7. The high concentration of NaCl was used to mimic the hypersaline conditions of the NaCl brines, and the KCl to provide an osmoprotectant for extreme halophiles.

After being mixed by inversion, unsterilized environmental brine samples were used to inoculate the media (500 μL aliquots). At the time of culturing the brines had been stored in the fridge at 4°C for <2 weeks. All aerobic enrichments were incubated at 37°C (typical optimum temperature for cultivating Halobacteriales, Rodriguez-Valera, 1995), at 80 rpm, and growth was examined using a combination of microscopy and optical density over a 30 day period. This temperature combined with the accessible carbon sources aimed to maximize the energetic favorability of cellular respiration to determine if the geochemistry of the brines limited microbial growth.

Brines that failed to demonstrate growth during the initial round of culturing were subject to an expanded range of experimental conditions. Environmental brine media were inoculated with the community from other brines (to test if certain brines were habitable, but did not contain any viable organisms), enrichment cultures grown from other environmental brine samples and soil (two types containing 10 g/L yeast extract, and 5 g/L Na pyruvate + 5 g/L casamino acids, both with 250 g/L NaCl) (see Table 2 for full list).

The range of artificial media was also expanded to include media mimicking the composition of the two brines that failed to demonstrate growth (101-P and 29XC), but with lower total ion concentrations, to test whether this parameter accounted for their inability to support growth. This included a MgCl_2_-rich nutrient and tryptic soy broth (with composition as follows: MgCl_2_ 250 g/L, KCl 2 g/L and tryptic soy broth 25 g/L or nutrient broth 19 g/L, at pH 3.5) to mimic 101-P, but with the MgCl_2_ levels reduced to 2 M (under the proposed MgCl_2_ limit to life, 2.3 M; Hallsworth et al., 2007). A high KCl nutrient and tryptic soy broth (KCl 200 g/L and tryptic soy broth 25 g/L or nutrient broth 19 g/L, both balanced to pH 7) was also created to mimic 29XC. Additionally, enrichments in HM media (Vreeland and Hochstein, 1992), a well-established media for enriching organisms from brines, were used to test for growth.

Some experiments using environmental brine media and artificial media were carried out under anaerobic conditions (see Table 2 and Supplementary Table 3) to broaden the growth conditions and maximize the chance of a positive enrichment. Triplicate 50 mL samples of each brine/carbon source mixture were added to 200 mL serum bottles, crimp sealed with rubber butyl stoppers and aluminum tops and sparged with nitrogen gas. After sparging, the bottles were amended with 0.8 mM of L-cysteine-HCl to reduce residual oxygen and the pH checked by removing an aliquot of brine. The serum bottles were then sterilized using an autoclave. Once cooled, 500 μL of the anoxic unsterile brine samples were injected as the inoculum and the media incubated at 37°C for 60 days.

### Brine Physicochemical Properties

Water activity was measured in the laboratory using a Rotronic HP23-AW-A water activity meter (Hauppauge, NY, United States). This was calibrated using five points of reference with water activities of 0.935, 0.845, 0.755, 0.595, 0.325 composed of saturated calibration solutions KH_2_PO_4_, KCl, NaCl, NH_4_NO_3_, MgCl_2_, respectively, as detailed in Winston and Bates (1960). Triplicate sample measurement and calibration took place in a 30°C incubator to ensure the temperature was kept constant.

An agar gelation assay was used to measure brine chao/kosmotropicity (see Cray et al., 2013). To improve on the efficiency of this methodology whilst retaining data quality, a Biotek Synergy 2 plate reader was used instead of a water bath and spectrophotometer. This adapted methodology involved adding 150 μL of preheated agar/filtered brine mixtures to a preheated 96 well-plate, and placed in the plate reader at 65°C where the temperature was decreased 1°C every 15 min and absorbance at 500 nm (A_500_) was recorded until it reached room temperature. As in Cray et al. (2013), an extra-pure reagent grade agar (Nacalai Tesque; Kyoto, Kansai, Japan) with a well characterized gel point of 43.5 ± 0.3°C (see Hallsworth et al., 2003) was used at a concentration of 1.5%. The gel point temperatures of agar/brine mixtures were determined by comparing increase in A_500_ as they cooled to that of a pure 1.5% agar solution. Once the mixture achieved the same increase in A_500_ as the pure agar at gel point (43.5°C), it was deemed to have undergone gelification.

Ionic strength has been identified as a potential physicochemical habitability limiter (Fox-Powell et al., 2016) due to its impact on the function and structure of biological macromolecules (e.g., Baldwin, 1996; Kunz et al., 2004). Ionic strength was also calculated for each of the brines from the ionic composition data collected using the equation below:

(1) $$I=0.5\sum c_{i}z_{i}^{2}$$

*c*_i_ = concentration of ion i (in mol liter^−1^)

*z*_i_ = charge of ion i

### DNA Extraction, Sequencing, and Bioinformatic Analyses

Ten liters of each brine were filtered using MO BIO 0.22 μm water filters attached to a peristaltic pump. These filters were then placed in a sterile 50 mL Falcon tube with flame sterilized tweezers. DNA extraction was challenging due to the abundance of clay minerals in the brines. A PowerMax Soil DNA Isolation Kit (QIAGEN; Hilden, North Rhine-Westphalia, Germany) with clay-proof modifications from Direito et al. (2012) was used. This involved placing the water filters in filter sterilized 15% ethanol/1 M phosphate buffer at pH 8 for the bead beating and lysis steps of the kit protocol and then incubating the tubes post-lysis in an 80°C water bath for 40 min. This method greatly enhanced DNA recovery. DNA was concentrated using an adapted protocol from Urakawa et al. (2010) (ammonium acetate and isopropanol), with the addition of linear acrylamide to aid recovery and pellet visualization. DNA extraction quality was checked throughout using PCR amplification (primers for bacteria were 27F and 1389R, and 21F and 958R for archaea) and electrophoresis gel as concentrations were too low for accurate NanoDrop Lite (Thermo Fisher) detection. Cell visualization and enumeration was trialed in the environmental samples, but proved extremely inconsistent due to amount of clay in the brines. Both bacteria and archaea specific PCR was carried out on all extractions, with DNA from *Escherichia coli* and *Haloferax volcanii* used as positive controls for bacteria and archaea, respectively, and to spike the brines to ensure their chemistry did not impact DNA recovery. Amplification products produced using archaeal primers were consistently more concentrated than those produced using bacterial PCR. Of the three brines able to have DNA extracted, the weakest PCR amplification was observed in 44XC, suggesting it had the lowest concentration of DNA.

DNA was sequenced using Illumina (San Diego, CA, United States) MiSeq for brine 215 and NextSeq for Billingham and 44XC. This diversity of techniques was related to the methods employed by the different funding bodies involved. MiSeq sequencing was carried out at Edinburgh Genomics, Edinburgh, United Kingdom and the NextSeq at Marine Biological Laboratory, Falmouth, United States. For NextSeq, libraries were constructed with the Nugen Ovation Ultralow Library protocol, targeting an insert size of 225–250 bp to enable read merging. Sequencing was then completed with a 2 × 150 paired-end run using dedicated read indexing. The library was prepared for MiSeq using a standard TruSeq DNA kit, targeting a 400 bp insert library. This was sequenced with a 2 × 250 bp paired end-run. Blanks were also sent for amplicon sequencing, but were failed by the sequencing center due to lack of DNA. Unassembled sequence files were then uploaded to MetaGenome Rapid Annotation with Subsystem Technology (MG-RAST), for taxonomic and functional analysis (Meyer et al., 2008; Keegan et al., 2016). SEED Subsystems and Kyoto Encyclopedia of Genes and Genomes (KEGG) mapping were used to examine the functional gene profile on the MG-RAST server, and RefSeq for the taxonomic diversity. Taxonomic compositions were confirmed by EMIRGE (Miller et al., 2011) and PhyloSift (Darling et al., 2014), which showed similar results to the MG-RAST profiles.

## Results

### Brine Geochemistry and Physicochemical Characteristics

All brines collected contained close to saturation levels of several chloride compounds (Table 1). Sulfate was the second most common anion, except for in 29XC where bromide was more abundant. The dominant cation in most of the brines was sodium, with 29XC again the exception with potassium most common. Magnesium content varied, with higher concentrations present in 29XC and 44XC and most extreme in 101-P. Brine pH ranged from near neutral in the two pools (Billingham and 215), to more acidic in 29XC and 44XC. The lowest pH recorded was 3.42 in 101-P. Dissolved oxygen (DO) in the brines varied between the brine pools. Billingham pool had the highest levels at 2 mg/l. Brine 215 and 101-P were lower at 1.1 and 0.8 mg/l, respectively. As mentioned, accurate DO measurements were not possible to obtain for 29XC and 44XC, although they are suspected to be suboxic/anoxic due to their depth and lack of exposure to the mine air. TOC/TIC measurements show that the brines from the taps contain concentrations of organic carbon slightly lower than typical rivers (TIC/TOC, 29XC 4.06/6.8 mg/l, 44XC 5.43/8.2 mg/l, 101-P 0.85/20.3 mg/l). The pressure in brine 44XC was measured at 58.5 psi (0.4 MPa). 29XC was significantly higher at 375 psi (2.6 MPa), and effervesced when sampled.

Billingham Baths and Brine 215 displayed very similar δ^2^H and δ^18^O values. These values were a little higher in Brines 29XC and 44XC. Brine 101-P, however, was the most enriched in ^2^H and ^18^O, with δ^18^O and δ^2^H values higher than any of the other brines (see Table 1).

Water activity for brines 29XC, 44XC, Billingham and 215 ranged from 0.72 to 0.742, but was lower in brine 101-P (0.566). Similarly, all the brines displayed similar kosmotropic behaviors, except 101-P which was distinctly chaotropic (positive heat capacity, see Table 1 and Supplementary Figure 1). The chaotropicity of Billingham Baths’ was not obtained due to its chemistry being nearly identical to 215. None of the brines were extreme in terms of ionic strength, with values between 5.3 and 6.6 mol/L.

### Culture-Dependent Analysis

**FIGURE 3:**
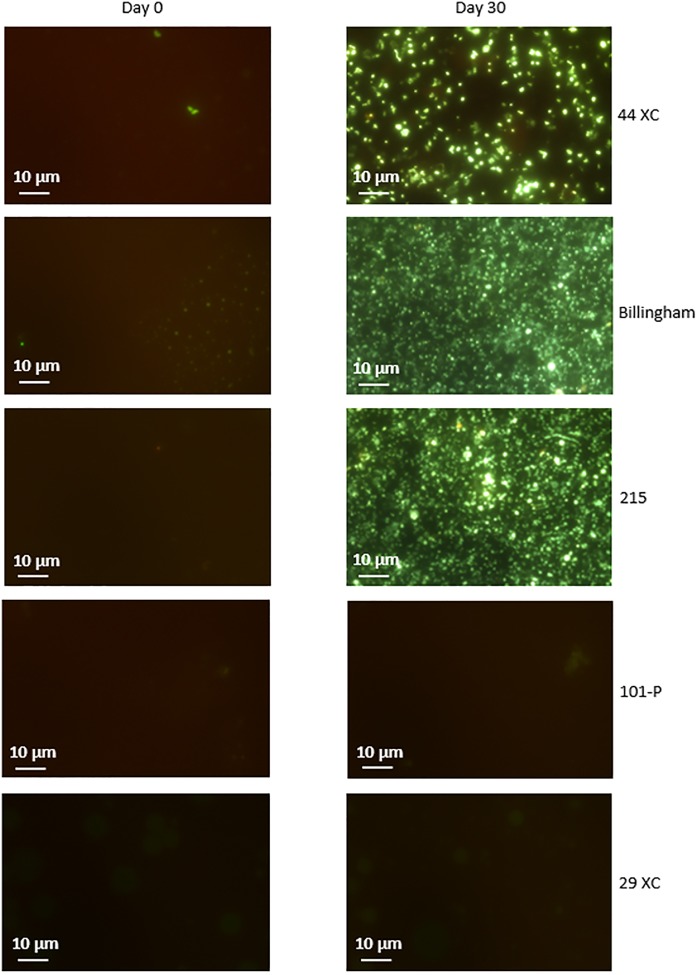
Microscopy of cultures in filtered brines containing nutrient broth inoculated with the same brines unsterilized. 44XC, Billingham and 215 all displayed dense enrichments after less than 30 days. 29XC and 101-P showed no growth. SYBR gold nucleic acid stain was used to visualize cells. Some images display regions of none specific stain binding to clay particles such as in 101-P.

Culturing using sterilized environmental brine samples with an added carbon source, produced rapid growth in 215, 44XC and Billingham, whilst 101-P and 29XC failed to produce growth (see Figure 3 and Supplementary Table 1). This pattern was repeated in the artificial media inoculated with brine samples, with both 101-P and 29XC failing to produce positive enrichments and 44XC, 215 and Billingham producing dense cultures (see Supplementary Table 2).

Since no positive enrichments were identified for 101-P and 29XC, both were subject to a second round of enrichment culturing using a larger range of media and inocula. Neither brine produced any positive enrichments across the new set of media tested (see Supplementary Table 3). Using the expanded range of inocula (which included soil as well as biomass from other brines) on the carbon-enriched versions of brines 29XC and 101-P (Table 2) did result in growth in 29XC. However, 101-P failed to produce any positive enrichments in any of the experiments.

**Table 2:** Results from enrichments carried out in brines 29XC and 101-P.

		+/− growth in the brines for each triplicate
Inoculant	Carbon source	29XC	101-P
215	Yeast	+/+/+	−/−/−
215	Na pyruvate and casamino acids	+/+/+	−/−/−
44XC	Yeast	+/+/+	−/−/−
44XC	Na pyruvate and casamino acids	+/+/+	−/−/−
Billingham	Yeast	+/+/+	−/−/−
Billingham	Na pyruvate and casamino acids	+/+/+	−/−/−
215, 44XC, Billingham, yeast media enrichment cocktail	Yeast	+/+/+	−/−/−
215, 44XC, Billingham, Na pyruvate and casamino acids media enrichment cocktail	Na pyruvate and casamino acids	+/+/+	−/−/−
Soil	Yeast	n/a	−/−/−
Soil	Na pyruvate and casamino acids	n/a	−/−/−
Billingham, 44XC, 215 cocktail (anaerobic)	Yeast	n/a	−/−/−
Soil (anaerobic)	Yeast	n/a	−/−/−

*Each condition was run in triplicate (− no growth, + growth, n/a not carried out).*

### Metagenomic Profile of Habitable Brines

Three metagenomes were successfully obtained from the habitable brines and raw data was uploaded to MG-RAST for quality trimming and annotation (Keegan et al., 2016) (MG-RAST ID’s 44XC, 4678909.3; Billingham, 4678908.3; 215, 4705070.3). DNA could not be obtained from 29XC or 101-P. The quality trimmed metagenome from brine 215 had a total size of 11.59 GB (gigabytes) composed of 16,139,625 sequences with a mean sequence length of 344 ± 75 bp (base pairs) and an average GC content of 60 ± 8%, Values for Billingham brine were: 4.02 GB, 16,279,907, 161 ± 26 bp, 64 ± 7% and 44XC brine: 4.69 GB, 11,049,776, 122 ± 49 bp, 57 ± 15%.

To compare the deep subsurface brines from this study with surface examples, six other publicly available metagenomes were examined and are mentioned in the results. Two of these metagenomes are from Bras del Port, Santa Pola, Spain (Rodriguez-Valera, MG-RAST ID’s 4441050.3, 4442451.3) and four from South Bay Salt Works, Chula Vista, CA, United States (Rodriguez-Brito, MG-RAST ID’s 4440438.3, 4440433.3, 4440429.3, 4440430.3).

**FIGURE 4:**
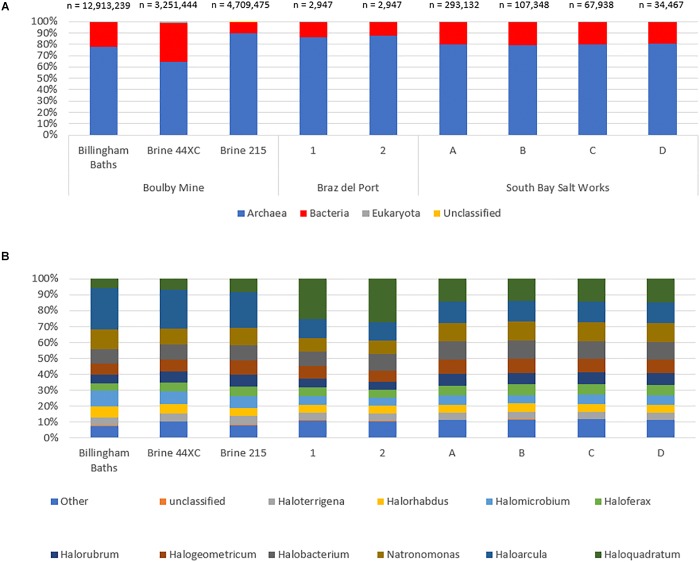
The taxonomic structure of the Boulby brines and a range of surface hypersaline brines as a percentage abundance by domain **(A)**, and top 10 genus in archaea **(B)**. Archaea dominate all three of the brines with bacteria making up a secondary portion of the brines. Eukaryotes and virus are the minority of sequences in all three. Note the different total sequence numbers for each metagenome.

At the domain level, the three habitable subsurface brines were dominated by archaeal sequences (Figure 4A), particularly from the order Halobacteriales (Figure 4B); a finding repeated in all six surface brines. Within this order, *Haloarcula* was the most abundant genera in the subsurface metagenomes (23% in 215, 26% 44XC and 26% in Billingham of Halobacteriales hits). Other common taxa included *Natronomonas* (215, 11%, 44XC, 10%, Billingham, 13%), *Halogeometricum* (215, 9%, 44XC, 8%, Billingham, 7%), *Halomicrobium* (215, 7%, 44XC, 8%, Billingham, 10%) and *Halobacterium* (215, 10%, 44XC, 10%, Billingham, 9%). In the surface brines, *Haloquadratum* was dominant, although it is worth noting that the metagenomes from Bras del Port were deliberately sampled to include high numbers of *Haloquadratum*. Despite some differences in relative abundance, the proportions of the most abundant genera between the surface and subsurface brines are comparable within the Halobacteriales order.

Bacterial populations were compositionally similar between the three subsurface brines, but differed somewhat in relative abundance. The most abundant bacteria phyla included Proteobacteria (215, 37%, 44XC, 66%, Billingham, 61% of bacterial hits), Bacteroidetes (215, 13%, 44XC, 5%, Billingham, 15%), Firmicutes (215, 17%, 44XC, 11%, Billingham, 7%), Actinobacteria (215, 14%, 44XC, 11%, Billingham, 7%), and Chloroflexi (215, 6%, 44XC, 1%, Billingham, 2%). Cyanobacteria appear to be present in both surface and subsurface metagenomes, but were more abundant in the surface metagenomes generally. Some of these hits, however, could be incorrectly assigned in our deep subsurface metagenomes as we did not see Cyanobacteria’s distinct 16S in our analysis. *Mycobacterium* and *Bradyrhizobium* were highest in 44XC, indicating low levels of contamination (see Discussion).

**FIGURE 5:**
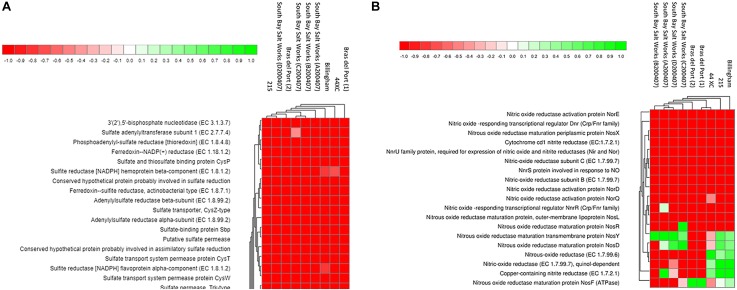
Heatmaps generated from the MG-RAST pipeline SEED Subsystems analysis of three brines collected during this study (44XC, Billingham and 215), and six other publicly available hypersaline brines, for denitrification **(A)** and inorganic sulfate metabolism **(B)** pathways. Data was normalized using DESeq package built into MG-RAST. Brightest green represents the most abundant features (1) and red, the least abundant (−1). For more detail on the generation of these plots, see Wilke et al. (2016).

In terms of functional genes, the brines were comparable (Figure 5). A total of 4,279,250, 1,402,652, and 424,227 hits were identified in the Subsystems database in the 215, Billingham and 44XC metagenomes, respectively. Housekeeping functional groups including protein (215, 10.06%, 44XC, 10.83%, Billingham, 10.36% of protein hits), amino acids (215, 9.99%, 44XC, 10.51%, Billingham, 11.12%), and carbohydrate (215, 9.22%, 44XC, 9.87%, Billingham 9.55%) metabolism were the most abundant. Focus here will be given to genes involved in metabolic processes. The pathways detailed below were identified using both Subsystems and KEGG. Comparing functional profiles between subsurface and surface brines using the DESeq package built into MG-RAST to normalize the data (Anders and Huber, 2010; Lin et al., 2016), also shows a range of similarities.

Denitrification is an anaerobic metabolism employed by several halophiles that function at NaCl saturation (Oren, 2011, 2013). Genes coding for enzymes involved in denitrification (nitrate reductase, nitrite reductase, nitric oxide reductase and nitrous-oxide reductase) were all present in the three metagenomes, associated with organisms from genera such as *Haloarcula*, *Halomicrobium*, *Halorhabdus*, and *Halogeometricum*. These genes were largely absent from the surface brines.

Fumarate reductase, a key enzyme in the reduction of fumarate, is present in the subsurface metagenomes, associated predominantly with *Halorubrum*. Genes coding for key enzymes involved in fermentative arginine degradation (ADI pathway) were found in the three metagenomes. These were attributed to organisms including *Salinibacter*, *Pseudomonas*, and *Halobacterium*. Enzymes involved in arginine production by fermentation and homolactic and ethanol fermentation were also found in the subsurface metagenomes and some of the surface brines.

Inferred carbon utilization pathways are also similar between the surface and subsurface brines. Pathways related to the metabolism of many monosaccharides, polysaccharides and amino acids were detected in the subsurface metagenomes, including complete pathways for galactose, fructose, mannose and xylose metabolism, assigned to *Halorhabdus*, *Halomicrobium*, *Chromohalobacter*, *Halomonas*, and alanine, arginine, glycine, isoleucine and valine metabolism related to *Natronomonas* and *Haloarcula*. These pathways were also present in surface metagenomes.

Genes coding for enzymes such as catechol 1,2-dioxygenase or protocatechuate 3,4-dioxygenase, which participate in the degradation of aromatic compounds (Bonfá et al., 2013), were only observed in our metagenomes in small numbers of bacterial hits from organisms such as *Pseudomonas*. Similarly, small numbers of hits related to alkane hydroxylases, important enzymes in the degradation of alkanes (Nie et al., 2014), were detected. Alkane 1-monooxygenase was observed in the subsurface metagenome KEGG results, related to bacteria such as *Pseudomonas*, except for in Billingham, where most of the hits were related to *Alcanivorax*, an important oil degrading organism (Golyshin et al., 2003). Key genes involved in the degradation of polycyclic aromatic hydrocarbons (PAHs), such as naphthalene dioxygenase were absent from the subsurface metagenomes and surface brines.

Another potentially available recalcitrant carbon source shown to exist in Permian rock salt is cellulose (Griffith et al., 2008). Endoglucanase and beta-glucosidase were present in the surface metagenomes South Bay Salt Works metagenomes (4440438.3, 4440429.3) and all subsurface metagenomes in the KEGG analysis. Hits in the subsurface came from *Halogeometricum*, *Natronomonas*, and *Haloarcula*.

Some genes related to carbon fixation are present in the metagenomes. RuBisCO is an important enzyme involved in photo- and chemoautotrophic CO_2_ fixation in a range of different proteobacteria (Badger and Bek, 2008), and has been found in other deep subsurface environments (e.g., Alfreider et al., 2009). RuBisCO (type III) was present related to organisms such as *Natronomonas* and *Halomicrobium*. RuBisCO was present in several of the South Bay Salt Works metagenomes. There is little evidence of functional genes related to other autotrophic pathways. For example, a key enzyme in the rTCA cycle, ATP citrate lyase, was absent.

## Discussion

### Origin of the Brines

**FIGURE 6:**
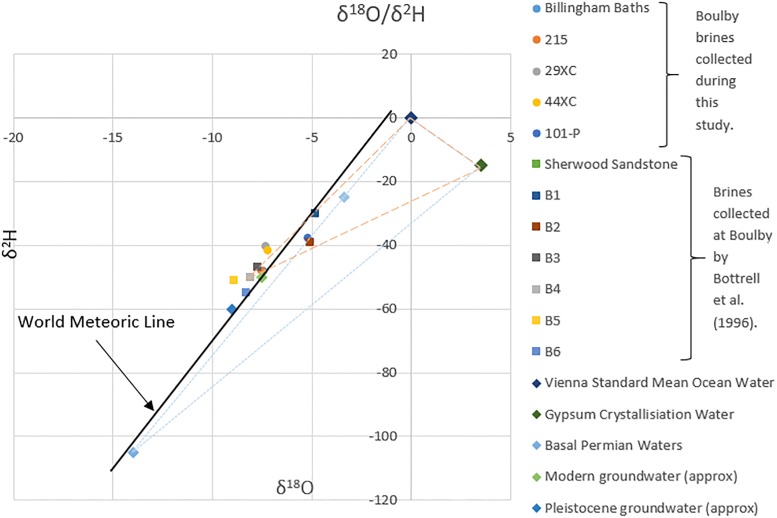
Isotopic data from the brines collected during this study and from Bottrell et al. (1996), plotted with the Global Meteoric Water Line, Standard Mean Ocean Water (SMOW), gypsum crystallization water and other British groundwaters. Two theoretical mixing fields have been added. Both draw a line between ocean water and gypsum crystallization water compositions (since gypsum water of crystallization isotopic composition will be some mixture of the two). The dashed line indicates a mixing of a gypsum crystallization water/ocean water, with meteorically derived groundwater (Sherwood Sandstone waters). The dotted line does the same, except with the isotopically light Permian groundwater taken from Sheppard and Langley (1984). Modern and Pleistocene ground water approximate values are from Darling et al. (2003).

Combining the brine isotopic and ionic compositions with regional geological and hydrogeochemical information makes it possible to infer their history. Billingham and 215 brines isotopically fall in the range of most modern groundwater found in Britain (approximately −8‰ to −6‰ δ^18^O and −55‰ to −30‰ δ^2^H from Darling et al., 2003), with some mixing with Pleistocene ground water (see Figure 6). These isotopic values, combined with their geochemical similarity to the Sherwood Sandstone waters collected by Bottrell et al. (1996), suggests they originated from the Sherwood Sandstone aquifer, dissolving halite during their passage through the evaporites. This hypothesis is consistent with the presence of a large fault associated with persistent brine influxes intersecting the mine where the brines were found. This fault likely acts as a conduit for fluids moving between the two geological units.

Both 29XC and 44XC appear to have more complex histories. The isotopic values of these two brines fall roughly in the range of most modern groundwater in Britain, suggesting they are related to the Sherwood Sandstone aquifer. However, higher levels of Mg^2+^, K^+^, and Br^−^, suggests interaction with a range of evaporite minerals between the aquifer and the mine. The high ratio of K^+^ to Na^+^ in brine 29XC is appears to be unusual compared to the Na^+^ dominated brines typically encountered in the mine.

The isotopic composition of brine 101-P lies outside the range expected for the regional modern groundwater. Bottrell et al. (1996) inferred that a similar shift in isotopic composition in their “group 3” brines could be explained by a mixing of Sherwood Sandstone groundwaters and “evaporite waters”; the latter referring to gypsum and other hydrous minerals waters of crystallization and interstitial sea water (see Figure 6). Although 101-P fits this profile, for evaporite waters to be contributing to the composition of brine 101-P, they would have had to been trapped since deposition, despite experiencing a maximum burial depth of >2.2 km during the Jurassic/Cretaceous (Talbot et al., 1982) which should have released the evaporite waters due to increased temperature and pressure. Therefore, based on the data collected, we find it hard to infer the exact source of 101-P, except that it is distinct from typical groundwaters in the region. Brine 101-P’s increased Mg^2+^ content does suggest interaction with the thick Mg-rich limestone layers close to the base of this Zechstein cycle.

### An Ionic Limit to Life

The culturing experiments demonstrate that the ability of the brines to support microbial growth varies. Organisms grew successfully in 44XC, Billingham and 215 when amended with carbon sources, showing these brines were habitable. In contrast, brine 101-P failed to produce growth in all enrichments attempted, suggesting it is uninhabitable. Brine 29XC was more complex. No organisms could be cultured from the brine. However, enrichment cultures within the brine were successful when using other brines as an inoculum, suggesting the brine is habitable, but does not contain viable organisms. This could be due to combined stressors of high pressure and high KCl concentrations, and a loss of some viable organisms during depressurization.

The reasons for these apparent differences in habitability can be elucidated by examining the physicochemical characteristics of the brines. The chao/kosmotrocity levels are theoretically permissive to life in all of the brines when factoring in recent work by Steinle et al. (2018). Brines 44XC, 215, and 29XC are all kosmotropic, with 29XC being the most kosmotropic due to its KCl content (Cray et al., 2013). In contrast, 101-P was chaotropic. Hallsworth et al. (2007), suggested chaotropicity equivalent to 2.3 M (or 124.2 kJ kg^−1^ as heat capacity) of MgCl_2_ was the upper limit for microbial propagation. This limit was raised by Steinle et al. (2018), who observed evidence of sulfate reduction occurring at > 300 kJ kg^−1^ in Kryos MgCl_2_-brine, a significant deviation from previous work on similar brine systems. 101-P increased heat capacity by 129.63 kJ kg^−1^, placing it beyond Hallworth’s hypothetical limit to life but within Steinle’s.

Brines 44XC, 215, 29XC and Billingham Baths all have water activities above the currently debated limits to biological activity in brines. At 0.566 a_w_,101-P again falls between the previous record for microbial propagation at low water activity of 0.585 a_w_ from Stevenson et al. (2017), and the significantly lower ∼0.4 a_w_ reported by Steinle et al. (2018). 101-P also has a low pH, which is not independently a barrier to life, but could act in concert with other stressors to restrict growth.

It is not possible to isolate either water activity, chaotropicity or any other physicochemical property as the limit to microbial propagation in 101-P. The brine is extreme with regard to several parameters that are well known to powerfully limit microbial growth. The ionic interactions that govern these physicochemical properties are currently not well understood. Additionally, the habitable regimes of water activity and chaotropicity are liable to change as more of the terrestrial biosphere is explored. For these reasons, we consider 101-P’s total ionic composition, which is responsible for its specific physicochemical profile, to be the barrier to microbial propagation, rather than any one individual physicochemical parameter.

Our results show that a range of deep subsurface fluids sampled over relatively small spatial distances can vary greatly in their ability to support life. All habitable brines originated from the Sherwood Sandstone aquifer. The only uninhabitable brine (101-P), originated, at least partly, from a separate source. High concentrations of Mg^2+^ and Cl^−^ typically induce low water activity and strongly chaotropic behaviors (Hallsworth et al., 2007), suggesting they are responsible the brine 101-P’s lack of habitability. The Mg^2+^ may have been acquired through interactions with the thick dolomitized limestone beneath the mine, a common feature of evaporite deposits. These dolomitized regions could be important for limiting microbial propagation in fluids within the Zechstein sequence.

It is worth noting that it would always be possible broaden the range of carbon sources, growth temperatures, inoculants and ionic compositions of the media to increase the chances of a positive enrichment. Additionally, many microorganisms appear not to be culturable with modern enrichment techniques, something that may be especially true when dealing with deep subsurface samples. This was in some way mitigated during these experiments by using communities of organisms from a number of different sources as opposed to isolates to better simulate the complexity of natural environments. Further work could strengthen our hypothesis that 101-P (and other similar brines) is uninhabitable, but realistically this can never be definitive.

### Habitable Brine Communities

The similarity of the communities between the surface and subsurface brines is striking. Generally, they contain similar proportions of archaea to bacteria and are dominated by the Halobacteriales order. Specifically, within this order there are some minor differences in dominant genera, but broadly the genera present between the surface and subsurface brines are very similar. It does not appear that genera such as *Halorhabdus*, which contains the hypoxic adapted halophile *Halorhabdus tiamatea*, were over represented in the subsurface samples relative to the surface (Antunes et al., 2008), despite the presumed hypoxic subsurface environment.

Similarly, the bacterial populations between the surface and subsurface are also generally alike at higher taxonomic levels. 44XC displayed a higher proportion of bacteria than the rest of the brines. This may be related to the different migration pathway taken by the brine through the subsurface, meaning it encountered and entrained different organisms from outside the evaporite deposit. However, it could also be related to minor contamination. Since 44XC appeared to contain the least DNA of the three habitable brines, it probably originally contained fewer organisms, therefore exaggerating any contamination present. The increased occurrence of organisms such as *Bradyrhizobium* in 44XC’s bacterial sequences in some way supports this (e.g., Salter et al., 2014), although these organisms are abundant in a number of environments. Whilst many controls and precautions were taken, low levels of contamination can only be mitigated rather than completely avoided, especially given the active mine context. As 44XC and 215 were both sampled directly from or very close to taps that had been accessing fresh brine seeps for over a year (thereby diluting an initial contamination), we believe the mine environment generally had little influence on community structure and composition of the brines analyzed. Billingham was exposed to the mine air for longer than 215 and 44XC, as it was allowed to pool for a short period of time, but its community is still compositionally very similar to 215 and 44XC.

The taxonomic and functional similarities between the deep subsurface brines (particularly 44XC which had not been exposed to the mine) and those found in extreme surface NaCl brines, suggests that even in the brines able to support microbial propagation, ionic composition still drastically limits the types of life present. In this way, NaCl at saturation levels appears to be a far stronger influence on community structure than any of the environmental differences between the surface and subsurface experienced by these brine communities (e.g., light, direct access to photosynthetically derived carbon, atmospheric oxygen).

Despite this limit imposed by NaCl, functional features of these halophilic communities enables them to deal with the additional environmental pressures imposed by the deep subsurface. As discussed, although we were not able to accurately sample the native brine dissolved oxygen content, we assume given the depth in which the brines are encountered they probably contained low levels of dissolved oxygen. However, no evidence was observed of a substantial increased presence of anaerobic organism in any of the subsurface brines relative to the surface. Most halophiles rely on aerobic respiration for energy acquisition and the abundance of cytochrome c oxidase related genes in the subsurface metagenomes supports this. Although we could not measure the native brine oxygen content, oxygen supplies are often limited in the deep subsurface meaning other metabolisms would be required where oxygen levels are low. If the deep subsurface brines are anoxic as assumed, then the Halobacteriales are likely using a variety of facultative processes to gain energy, such as denitrification, fermentation and the reduction of fumarate. Consistent with this hypothesis is our observation that denitrification genes related to *Haloarcula*, *Halomicrobium*, *Halorhabdus*, and *Halogeometricum* are found in the subsurface brines, but are absent from the surface metagenomes. Despite generally being scarce in hypersaline environments (Oren, 2013), it is worth noting that nitrate was detected in 44XC, showing it is present in certain brines in the deposit and therefore could be available for use.

TIC/TOC values in the brines are high for typical ground waters, particularly in 101-P where its chaotropic properties might act to preserve organic matter (Hallsworth et al., 2007; Polymenakou et al., 2007). This could provide an electron donor for the communities present in the brines which are shown in the metagenomes to be capable of using a range of carbon sources, including cellulose which has been shown to exist in Permian rock salt (Vreeland et al., 1998; Griffith et al., 2008). Whilst the potential presence of autotrophic cyanobacteria and RuBisCO in the deep subsurface metagenomes is consistent with other deep subsurface metagenomes (e.g., Kormas et al., 2003; Miettinen et al., 2015), their role in deep subsurface carbon production is still unclear (Alfreider et al., 2009; Miettinen et al., 2015).

It is worth noting that the failure to detect certain genes in a metagenome does not exclude a metabolism being present in that environment. However, to speculate, the dominance of chemoheterotrophs, a general lack of evidence for autotrophic metabolisms (outside of RuBisCO), and the absence of genes related to many parts of the nitrogen and sulfur cycles (mirroring similar results from other surface halophilic communities, Fernández et al., 2014, 2015) may indicate that the habitable brine communities only have a narrow function in the general cycling of carbon and elements in the deep subsurface. Ultimately they may rely on the production of energetically useful chemical species in less extreme adjacent geological environments and buried photosynthetically fixed carbon.

## Conclusion

These data demonstrate the influence fluid ionic composition can have on defining limits to life in the deep subsurface. Changes in ionic composition, possibly due to interaction with dolomitized limestone, were shown to result in some brines being unable to support microbial propagation. The widespread presence of Mg^2+^/Ca^2+^/Cl^−^ bearing minerals in evaporite sequences suggests that other parts of the deep subsurface are likely rendered uninhabitable at depths where temperature and pressure are not significant barriers to life. The microbial communities present in the habitable brines closely resembled the halophilic communities present in several hypersaline surface environments. This indicates that even in the brines able to support microbial propagation, ionic composition still limits the community present despite the environmental differences between the surface and subsurface. Overall this work highlights how ancient evaporites laid down in the Permian continue to shape the microbial deep biosphere.

## Data Availability

The datasets generated for this study can be found in MG-RAST, 4678909.3, 4678908.3, 4705070.3.

## Author Contributions

SP led the project supervised by CC (primary) and BN (secondary). JB advised on the metagenomic work and contributed part of the data analysis. BSL advised on and contributed to the isotopic analysis. MF-P advised on the physicochemical analysis and assisted in the field work. SP and TE helped to organize and support the field work.

## Conflict of Interest Statement

The authors declare that the research was conducted in the absence of any commercial or financial relationships that could be construed as a potential conflict of interest.

## Supplementary Material

FIGURE S1 Change in gel point temperature plotted against brine concentration in agar. The addition of brines altered the agar gelation temperature significantly in the four brines tested. 29XC, 44XC, and 215 are shown to increase the gel point temperature of the brines, whilst 101-P decreases it significantly. Triplicate values represent experimental rather than measurement triplicates. *R*^2^-values are consistently above 0.95.

TABLE S1 Results of attempts at enriching organisms using sterilized environmental brine samples with a carbon source added as media to test habitability after 30 days of enrichment at 37°C. Each condition was done in triplicate (− no growth, + growth). This experiment amended filter sterilized brines using two sets of carbon sources (yeast extract and Na^+^ pyruvate and casamino acids) and used the same brines, but unsterilized, to inoculate the carbon amended brines. 215, 44XC and Billingham are all clearly habitable and rapidly became opaque with high densities of halophiles. 29XC and 101-P both failed to produce any kind of microbial enrichment.

TABLE S2 Results of initial attempts at enriching organisms in two media types to test habitability after 30 days of enrichment at 37°C. Each condition was done in triplicate (− no growth, + growth). 215, 44XC and Billingham consistently produced growth when used as an inoculum in these two media types. Both 29XC and 101-P failed to produce microbial growth. One of the 44XC triplicates did not produce any growth.

TABLE S3 Results from expanded set of culturing using brines 29XC and 101-P as inoculum. Each condition was done in triplicate (− no growth, + growth). Neither brine could produce a positive enrichment across the media tested. Anaerobic cultures were grown for 60 days and aerobic cultures 30 days, both at 37°C.
