# Supplementary figures and images for: An Ionic Limit to Life in the Deep Subsurface
Source: Front Microbiol. 2019 Mar 12;10:426. doi: 10.3389/fmicb.2019.00426 (PMC6422919; doi:10.3389/fmicb.2019.00426)

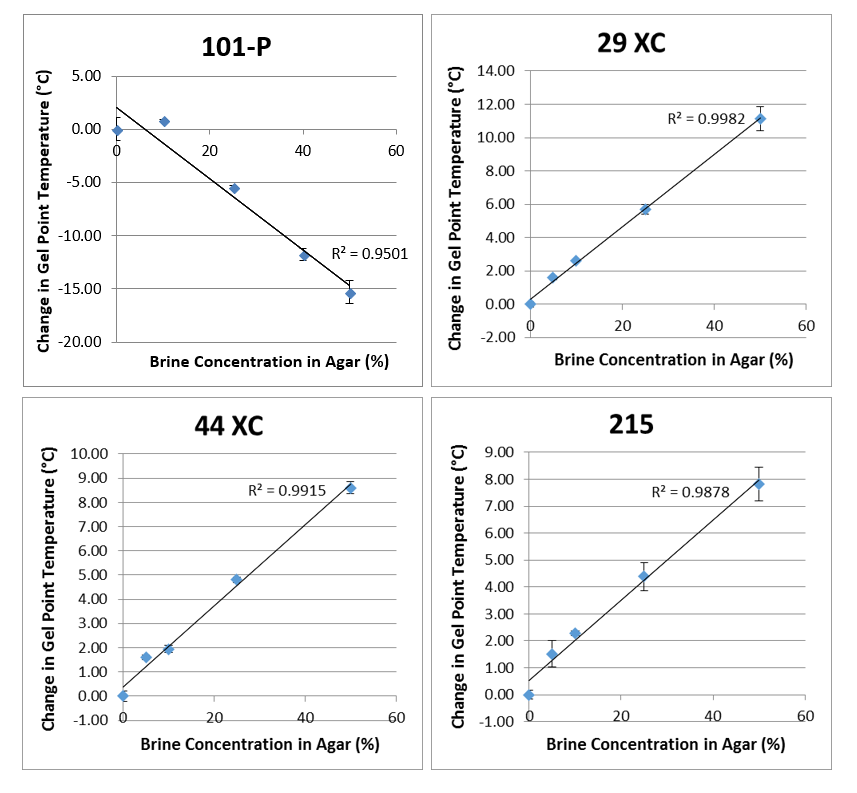

Supplement: FIGURE S1 — Change in gel point temperature plotted against brine concentration in agar. The addition of brines altered the agar gelation temperature significantly in the four brines tested. 29XC, 44XC, and 215 are shown to increase the gel point temperature of the brines, whilst 101-P decreases it significantly. Triplicate values represent experimental rather than measurement triplicates. R2-values are consistently above 0.95. [file Image_1.TIF]
