# Supplementary material for: An Ionic Limit to Life in the Deep Subsurface
Source: Front Microbiol. 2019 Mar 12;10:426. doi: 10.3389/fmicb.2019.00426 (PMC6422919; doi:10.3389/fmicb.2019.00426)
Supplement: TABLE S1 — Results of attempts at enriching organisms using sterilized environmental brine samples with a carbon source added as media to test habitability after 30 days of enrichment at 37°C. Each condition was done in triplicate (− no growth, + growth). This experiment amended filter sterilized brines using two sets of carbon sources (yeast extract and Na+ pyruvate and casamino acids) and used the same brines, but unsterilized, to inoculate the carbon amended brines. 215, 44XC and Billingham are all clearly habitable and rapidly became opaque with high densities of halophiles. 29XC and 101-P both failed to produce any kind of microbial enrichment. [file Table_1.DOCX]

|  |  | **+/- growth in the brines for each triplicate** | | | | |
| --- | --- | --- | --- | --- | --- | --- |
| **Inoculant** | **Carbon Source** | **215** | **44XC** | **Billingham** | **29XC** | **101-P** |
| Unsterilised brine | Yeast | +/+/+ | +/+/+ | +/+/+ | -/-/- | -/-/- |
| Unsterilised brine | Na pyruvate and casamino acids | +/+/+ | +/+/+ | +/+/+ | -/-/- | -/-/- |

**Supplementary Table 1**
