# Supplementary material for: An Ionic Limit to Life in the Deep Subsurface
Source: Front Microbiol. 2019 Mar 12;10:426. doi: 10.3389/fmicb.2019.00426 (PMC6422919; doi:10.3389/fmicb.2019.00426)
Supplement: TABLE S2 — Results of initial attempts at enriching organisms in two media types to test habitability after 30 days of enrichment at 37°C. Each condition was done in triplicate (− no growth, + growth). 215, 44XC and Billingham consistently produced growth when used as an inoculum in these two media types. Both 29XC and 101-P failed to produce microbial growth. One of the 44XC triplicates did not produce any growth. [file Table_2.DOCX]

|  | **Inoculum** | | | | |
| --- | --- | --- | --- | --- | --- |
| Media | **215** | **44XC** | **Billingham** | **29XC** | **101-P** |
| High NaCl + KCl nutrient broth | +/+/+ | +/+/+ | +/+/+ | -/-/- | -/-/- |
| High NaCl + KCl tryptic soy broth | +/+/+ | +/+/- | +/+/+ | -/-/- | -/-/- |

**Supplementary Table 2**
