# Supplementary material for: An Ionic Limit to Life in the Deep Subsurface
Source: Front Microbiol. 2019 Mar 12;10:426. doi: 10.3389/fmicb.2019.00426 (PMC6422919; doi:10.3389/fmicb.2019.00426)
Supplement: TABLE S3 — Results from expanded set of culturing using brines 29XC and 101-P as inoculum. Each condition was done in triplicate (− no growth, + growth). Neither brine could produce a positive enrichment across the media tested. Anaerobic cultures were grown for 60 days and aerobic cultures 30 days, both at 37°C. [file Table_3.DOCX]

|  | **Inoculum** | |
| --- | --- | --- |
| Media | **29XC** | **101-P** |
| Aerobic high MgCl nutrient broth | -/-/- | -/-/- |
| Aerobic high MgCl tryptic soy broth | -/-/- | -/-/- |
| Aerobic HM Media | -/-/- | -/-/- |
| Aerobic high KCl nutrient broth | -/-/- | -/-/- |
| Aerobic high KCl tryptic soy broth | -/-/- | -/-/- |
| Anaerobic high MgCl nutrient broth | -/-/- | -/-/- |
| Anaerobic high MgCl tryptic soy broth | -/-/- | -/-/- |
| Anaerobic HM Media | -/-/- | -/-/- |
| Anaerobic high KCl nutrient broth | -/-/- | -/-/- |
| Anaerobic high KCl tryptic soy broth | -/-/- | -/-/- |

**Supplementary Table 3**
